# Targeted metabolomic and gene expression analysis reveals an organ-specific response to chronic ionizing radiation exposure during *Pinus sylvestris* seedling development

**DOI:** 10.1186/s12870-026-09149-7

**Published:** 2026-06-04

**Authors:** Brix De Rouck, Leen Janssen, Gustavo Turqueto Duarte, Els Prinsen, Nele Horemans

**Affiliations:** 1 Biosphere Impact Studies, Belgian Nuclear Research Centre (SCK CEN), Boeretang 200, Mol, Belgium; 2 Laboratory for Integrated Molecular Plant Physiology Research (IMPRES), University of Antwerp, Groenenborgerlaan 171, Antwerp, Belgium; 3 Centre for Environmental Sciences (CMK), Hasselt University, Agoralaan, Diepenbeek, Belgium

**Keywords:** Pinus sylvestris, Ionizing radiation, Shoot apical meristem, Phytohormones, Amino acids, Senescence, Stress response, Methionine

## Abstract

**Background:**

Conifers like Scots pine (*Pinus sylvestris*) are a potential bioindicator species for radioactive contamination as they are among the most radiosensitive plant species. Phytohormones and amino acids are central to plant growth and stress signaling, but their roles in the pine radiation response, particularly in a tissue-specific context, have not been adequately studied.

**Results:**

Here, we investigated the morphological, metabolic and transcriptional consequences of chronic gamma irradiation in pine seedlings under controlled laboratory conditions. We exposed *P. sylvestris* seedlings to 10 weeks of low-dose external gamma irradiation (682 µGy/h) and performed targeted metabolomic analysis of phytohormones and amino acids and RT-qPCR analysis of hormone-related gene expression in the shoot tip, root tip, young needles, and cotyledons, alongside a microscopic analysis of the shoot apical meristem. Irradiation induced a significant reduction in the height and area of the meristem. This morphological change coincided with a trend towards decreased abundance of growth-promoting hormones in the shoot tip, namely cytokinins and gibberellins. Our data also indicate the potential involvement of specific cytokinin and gibberellin signals in the young needles in the response to irradiation, and hint towards a tissue-specific role for jasmonyl-ACC conjugation in the young shoot. Concurrently, the cotyledons of irradiated seedlings displayed a massive and significant increase in free amino acid concentrations and decreased cytokinin abundance, consistent with accelerated senescence.

**Conclusions:**

Taken together, these results reveal a tissue-specific pattern of metabolic and transcriptional changes in irradiated pine seedlings. The data are consistent with a scenario in which apical growth is hormonally suppressed and resources may be liberated from cotyledon tissue, which could then potentially be reallocated toward a stress response in developing organs. This study thereby provides an integrated view of the organ-level metabolic response to chronic radiation.

**Supplementary Information:**

The online version contains supplementary material available at 10.1186/s12870-026-09149-7.

## Background

Ionizing radiation is a physical phenomenon involving energetic particles or electromagnetic waves with enough energy to remove electrons from atoms or molecules. Radiation-induced ionization can be directly harmful to living organisms when it is imposed on important biomolecules such as DNA, proteins and lipids, while ionization of water molecules in living cells can lead to indirect damage due to the production of harmful reactive oxygen species through so-called water radiolysis [1]. Radiation sensitivity changes across taxa, but plants are generally regarded as more tolerant compared to animals, including humans [2]. An important exception to this rule are conifers, whose radiation sensitivity is comparable to that of mammals [2]. Indeed, conifers such as pine trees (*Pinus* spp.) have long been considered among the most radiosensitive plant species [3], a fact which is attributed to the abundance of genetic material present in their cells and a limited molecular response to irradiation [4, 5]. Hence, pine trees are regarded as promising bioindicator species in the context of radioactive pollution and they are used as reference species by the International Commission on Radiological Protection for estimating environmental impact of radiation [6, 7]. Field studies conducted in the area contaminated by the Chornobyl nuclear accident have revealed a variety of radiation-induced effects imposed on the local pine populations, even at radiation levels only slightly above the background and generally considered too low to induce any meaningful effects in plants [7, 8]. At these low-dose gamma levels, pine trees were affected at the genetic [9, 10], epigenetic [11–13], transcriptomic [14], metabolic and (sub)cellular level [15]. Furthermore, radiation exposure has been shown to induce the loss of apical dominance in pine trees growing in the Chornobyl-affected area [15, 16], as well as in pines and other conifers affected by the Fukushima nuclear accident [17, 18]. The latter has been attributed to changes to the abundance of plant hormones following radiation exposure [19].

Phytohormones are small organic signaling molecules that operate at minute concentrations to coordinate plant growth, development, defense, and physiological mechanisms across vascular and non-vascular species. They function as crucial messengers that integrate internal developmental cues with external environmental signals [20–22]. Over a dozen groups of phytohormones have been identified, with auxins, cytokinins (CKs) and gibberellins (GAs) being recognized as primary regulators of plant growth and development. Auxins, often indole derivatives derived from amino acids like Trp, are fundamental regulators of processes such as cell division, elongation, differentiation, apical dominance, and tropisms [23]. Cytokinins, which are mainly adenine analogues derived from isoprenoids, control cell division, maintain meristem activity, modulate root architecture, and delay leaf senescence [24–26]. Both auxin and CKs are the principal regulators of apical dominance, the mechanism by which the growth of the primary shoot apex inhibits the outgrowth of lateral axillary buds [27]. Auxin (specifically indole-3-acetic acid), synthesized predominantly in the shoot apex and present in the stem along a polar auxin gradient, acts as the core inhibitor of axillary bud outgrowth [27–29]. This inhibitory effect is indirect, mediated mainly by auxin repressing the local biosynthesis of CKs within the nodal stem [30, 31]. Conversely, CKs promote cell division and directly stimulate axillary bud outgrowth, acting antagonistically to auxin [27, 28]. Gibberellins, classified as tetracyclic diterpenoid acids synthesized from isoprenoids, are also major growth-promoting molecules involved in seed germination, stem elongation, and flowering [32, 33]. Plant growth is further controlled by brassinosteroids, which promote cell division, elongation, vascular differentiation and confer stress tolerance [34], and strigolactones, which are terpenoid lactones derived from carotenoids [35] and are responsible for regulating shoot branching and root development [36, 37]. Strigolactones reinforce the auxin-induced repression of bud outgrowth whereas auxin promotes the biosynthesis of strigolactones which in turn antagonize CKs by inhibiting CK production or converging on negative growth regulators like the transcription factor BRC1 [29, 37, 38]. Hence, the final interplay integrates apical auxin and strigolactone signalling, which regulate local CK activity, with growth signals mediated by GA and brassinosteroids.

Additional hormones act mainly as orchestrators of environmental stress responses, a crucial role for sessile organisms such as plants which are unable to escape a harmful or suboptimal environment. Abscisic acid (ABA), a sesquiterpene derived from isoprenoids [35], is recognized as a quintessential stress hormone, controlling seed dormancy, stomatal closure, and plant resilience to abiotic stresses such as drought and salinity [20, 39, 40]. Ethylene is another canonical stress hormone, synthesized from the amino acid Met via the precursor 1-aminocyclopropane-1-carboxylic acid (ACC) [41]. Ethylene regulates various developmental processes like fruit ripening, senescence and abscission [42–45], and is heavily involved in the plant stress response [45–47]. Jasmonates (JAs), which are oxylipin derivatives derived from lipids like $\:\alpha\:$-linolenic acid, mediate defense responses against pathogens and herbivores as well as adaptation to abiotic stresses like cold, drought, and salinity [48, 49]. Salicylic acid, a phenolic organic acid often derived from phenylalanine, is crucial for activating plant defense responses such as systemic acquired resistance, and plays a role in regulating photosynthesis and senescence [20]. Additionally, polyamines, which are aliphatic nitrogenous bases synthesized from amino acids, are typically regarded as growth regulators but are also important for enhancing tolerance to various abiotic and biotic stresses [22, 35, 50]. None of these phytohormones operate in isolation but through intricate cross-talk networks that are vital for sensing signals and orchestrating a comprehensive response, balancing growth and survival in challenging environments [51, 52].

While phytohormones provide pivotal control over plant growth and development, other metabolites are equally crucial. Amino acids, for example, serve primarily as the building blocks for proteins, mediating cellular pH control, and generating metabolic energy or redox power, especially under stress conditions [53, 54]. Their concentrations change dynamically based on developmental and physiological states [54], and many actively participate in plant defense against stress [55]. Beyond protein synthesis, amino acids are indispensable precursors for numerous specialized compounds. For example, Met is the immediate precursor for S-adenosyl-L-methionine, which serves as the major methyl donor for methylation reactions on e.g., DNA, histones, pectin and lipids and as a precursor for crucial plant hormones like ethylene and polyamines, as well as nicotinamine [56–59]. Aromatic amino acids like Phe and Trp are precursors for phenylpropanoids, some alkaloids, and glucosinolates [60, 61], while Trp is the precursor for the auxin indole-3-acetic acid [23]. Many amino acids (e.g., Lys, branched-chain amino acids, Thr, Phe, Trp) accumulate during abiotic stresses (e.g., drought, cold, high light intensity or prolonged darkness), functioning as alternative respiratory substrates to generate energy when resources are limited [54, 60, 62]. Proline and gamma-aminobutyric acid (GABA) also accumulate under stress, acting as important osmolytes (compatible solutes) to maintain osmotic balance and protect cellular structures, in addition to being ROS scavengers [53, 63, 64]. The metabolism and levels of amino acids are intricately regulated by metabolite transport, feedback inhibition, transcriptional programs responsive to environmental cues, and interactions with hormones [60, 61, 64, 65].

Despite the central role of plant hormones and amino acid homeostasis in growth regulation and stress responses, few studies have investigated the effect of abiotic stress on metabolomic changes in pine species [66–69]. Furthermore, metabolomic research in pines suffering from chronic radiation exposure has been limited to field studies [70, 71] which although bringing an environmentally relevant footprint inherently suffers from confounding factors [72]. Thus far, an in-depth analysis of phytohormonal changes due to irradiation in laboratory conditions has only been conducted in the model plant *Arabidopsis thaliana* [73]. While several metabolomic studies have allowed the investigation of the pine amino acid profile after abiotic stressors such as drought and heavy metal stress [74–76] no such analysis has previously been conducted in pine trees exposed to ionizing radiation. Given the hormonal basis proposed for radiation-induced morphological abnormalities in field-exposed pines, a controlled laboratory approach is needed to disentangle radiation-specific hormonal effects from the confounding factors inherent to field studies, and to obtain organ-level resolution that field sampling cannot provide. We therefore hypothesized that chronic gamma irradiation would induce tissue-specific changes to the phytohormone and amino acid profiles of *P. sylvestris* seedlings, and that these changes would be consistent with altered growth regulation in meristematic tissues and a stress-associated response in developing organs. To test this, we conducted a controlled laboratory experiment investigating the effects of chronic gamma irradiation on Scots pine seedlings. We performed a targeted, tissue-specific metabolomic analysis of key phytohormones and amino acids, linking these molecular changes to morphological alterations in the shoot apical meristem (SAM). In addition, we evaluated gene expression analysis on a select panel of hormone-related genes to further probe the hormonal response of pine trees to ionizing radiation.

## Methods

### Pine seedling culture, irradiation treatment and sampling

Scots pine (*Pinus sylvestris* L.) seeds of Dutch origin (region of provenance NL.ZQ.3.5.10–10, Grubbenvorst, The Netherlands), harvested in 2017 and certified for forestry use, were acquired from a commercial vendor (Pelgrum-Vink Materialen B.V., Lobith, The Netherlands). Pine seedlings were hydroponically cultured and irradiated as previously described [77]. Briefly, seeds were surface sterilized, germinated on moist vermiculite for two weeks and then cultured on the same vermiculite supplied with an aerated half-strength Hoagland solution [78] for another 8 weeks. Germination and seedling culture occurred in a climate chamber kept at 30% humidity and 24 °C/20°C (day/night) under a 16/8 h photoperiod at 140 µmol⋅m⁻²⋅s⁻¹ PAR. Seedlings in the treatment group were continuously exposed to gamma radiation from ^137^Cs sources placed adjacent to the seedlings starting at the moment of sowing, yielding a dose rate of 682 ± 74 µGy⋅h⁻¹ at shoot level, with mock sources placed next to seedlings in the control group. After 10 weeks of growth, 100 seedlings were phenotypically analyzed per condition. The number of axillary buds and axillary branches (defined as a growing bud with visibly discernible needles) formed by each seedling were counted and the shoot was removed and weighed. Tissues were then sampled for molecular analysis and immediately flash-frozen in liquid nitrogen. Non-meristematic tissues (cotyledons and young needles) were sampled separately from each seedling (*n* = 100 per tissue per condition), with the cotyledon sample consisting of all six cotyledons of the individual (ca. 30 mg of material) and the young needle sample consisting of 10–12 of the youngest needles (ca. 100 mg of material) of the individual. Meristematic tissues (root and shoot tips) were pooled to generate sufficient material for molecular analyses. Root tips (ca. 1 cm long) from eight separate seedlings were pooled into a single tube to constitute one biological replicate (*n* = 12 per condition). Similarly, shoot tips (ca. 0.5 cm) were harvested after removal of the young needles, with tips from eight seedlings pooled to form one biological replicate (*n* = 12 per condition; ca. 30 mg per pool). For each condition, five replicates of each of the non-meristematic tissues and six replicates of each of the meristematic tissues were used for metabolomic analysis. In parallel, five replicates of each tissue were used for gene expression analysis. In addition, five shoot tips per condition were set aside for microscopic analysis. The frozen samples were powdered by placing five to six 3 mm glass beads in the Eppendorf tubes and grinding the tissue twice using a Retsch^®^ MM 400 cryomill at 30 Hz for 2 min with the tube holders pre-chilled in liquid nitrogen.

### Microscopic analysis

Shoot tips were placed in 1% Triton X-100 (v/v) solution for 10 min, rinsed with distilled water and fixed with 3% (w/v) glutaraldehyde, 0.1 M sodium cacodylate in distilled water for 48 h at 4 °C. The fixed meristems were then dehydrated using an ethanol dilution series ranging from 30% (v/v) to 100% over the course of three days. The dehydrated samples were cleared by incubation in a 1:1 ethanol:xylene (v/v) solution for one hour followed by incubation in 100% xylene for 48 h. After clearing, the shoot tips were embedded in paraffin and cut into 7 μm sections using a microtome (Microm HM 340 E, Walldorf, Germany). The sections were placed on microscopy slides and dried, followed by deparaffinization and rehydration using a range of xylene and ethanol solutions. The slides were then stained by a 1 min incubation in 0.1% toluidine blue following 20 min of incubation in 1 M HCl at 60 °C, and coverslips were fixed to the slides using a drop of dibutylphthalate polystyrene xylene. The sections were imaged using a Nikon AZ100 microscope (Nikon Instruments Europe BV) with a Nikon AZ Plan Fluor 5x objective on 4x magnification. Pictures were processed with the NIS Elements D Imaging software (Nikon Instruments Europe BV) and the analysis of meristem dimensions was conducted using ImageJ version 1.54d as previously described [79] after anonymization.

### Metabolomics analysis

A targeted metabolomics analysis was conducted using ultra-high performance liquid chromatography coupled with tandem mass spectrometry (UPLC-MS/MS). The analyzed compounds were free and conjugated indole-3-acetic acid (IAA), isoprenoid and aromatic CKs, bioactive and precursor GAs, JA, ABA, ACC, and amino acids. Metabolites were extracted by addition of 600 µl of extraction solvent (20% 0.8 mM formic acid (v/v) in methanol) to the ground, frozen samples followed by brief vortexing, 10 min of sonication in an ice bath (45 kHz, 180 W, VWR ultrasonic cleaner) and overnight extraction in the dark at -20 °C. Heavy-isotope labeled tracer molecules (Supplementary Material A1) were added to the extracts prior to analyte purification to allow for quantification of target molecules. The different compounds were then purified as previously described [80]. Following analyte purification, all samples were analyzed using an ACQUITY UPLC System combined with an ACQUITY TQD tandem quadrupole UPLC/MS/MS. (Waters, Milford, MA, USA), with parameters depending on the targeted analytes (Supplementary Material A2, Tables A1-A8). The samples were injected into a VanGuard pre-column (BEH C18; 1.7 μm; 2.1 × 5 mm; Waters, Milford, MA, USA) coupled to a reversed phase column (BEH C18; 1.7 μm; 2 ,1 × 50 mm; Waters, Milford, MA, USA) for most hormones or a Waters ACQUITY UPLC^®^ Bridged Ethylene Hybrid (BEH) Amide column for ACC and amino acids. Quantification of the different compounds was carried out as described elsewhere [80]. ES+- MRM was used for detection of the analytes and TargetLynx™ 4.2 (Waters, Milford, MA, USA) was used to process the obtained chromatograms. We tried to define two specific transitions per compound of interest, the most dominant transition is used for quantification, whereas the second transition is used for confirmation. Retention times were evaluated using pure reference compounds. The actual endogenous concentrations in the samples were calculated following the principle of isotope dilution and expressed in pmol.mg^− 1^ fresh weight. For each compound, concentrations were then expressed as a log_2_ fold change relative to the mean of the control.

### Gene expression analysis

Gene expression analysis was carried out via RT-qPCR as previously described [77] with two technical replicates per reaction. After averaging technical replicates, relative expression of treated samples relative to the control was calculated within each tissue using the 2^−ΔΔCt^ method [81] with normalization to the geometric mean of all reference gene Ct values [82], and data were log_2_ transformed prior to statistical analysis. Utilized reference genes were identified from a panel of 9 candidate reference genes as having the most stable expression across root- and shoot tissues and across treatment conditions using RefFinder [83], a web tool providing integrative evaluation of reference gene stability by aggregating the results of popular reference gene analysis algorithms such as geNorm [82], NormFinder [84], BestKeeper [85] and a comparative C_t_ method [86].

RT-qPCR primers for *RR1* were designed using the published sequence of *RR1* as identified in *Pinus pinea* [87] (GenBank: FJ717710.1) while primers for actin were designed using the published sequence of *ACT1* as identified in *P. sylvestris* (GenBank: FN546174.1). Primers for other transcripts were designed using nucleotide sequences obtained from a *P. sylvestris* [14] or *P. densiflora* [88] (for *YLS8* and *PEX4*) reference transcriptome through maximal sequence homology to the *Arabidopsis thaliana* reference coding sequence. Primer specificity was assessed through NCBI Primer-BLAST using the *P. sylvestris* transcriptome [14] as a custom database and through melting curve analysis following RT-qPCR. Primer sequences and primer efficiencies are provided as Supplementary Material A2, Table A10.

### Statistical analysis

All statistical analysis was carried out in RStudio 2023.03.1 using R version 4.3.0. The shoot fresh weight, the number of buds, the number of outgrown branches and the percentage bud outgrowth (number of branches / (number of buds + number of branches)) were compared using Wilcoxon’s rank-sum test due to a violation of the assumption of normality. The dimensions of the apical dome were also compared between conditions using Wilcoxon’s rank-sum test due to violations of the assumptions for parametric testing. The distribution of the number of buds and branches was compared between the two conditions using Fisher’s exact test. For the gene expression analysis, log_2_ relative expression values relative to the mean of the control were compared between the control and treatment conditions for each gene and tissue using Wilcoxon’s rank-sum test. Because the RT-qPCR analysis was restricted to a targeted, a priori selected panel of seven specific genes, p-values were not adjusted for multiple testing. We treated these comparisons as independent, hypothesis-driven observations rather than a screening, prioritizing the limitation of false negatives (Type II errors) within this heavily focused dataset. For the metabolomics analysis, missing values due to samples with concentrations below the detection limit were imputed within each tissue as half of the lowest concentration value detected for the compound if the compound was detectable in at least half of the samples of that tissue for at least one of the two conditions, otherwise the compound was excluded from the analysis for that tissue. Outliers were removed according to the box plot method (removal of values above Q3 + 1.5 x IQR or below Q1–1.5 x IQR) as implemented in the *rstatix* R package version 0.7.2, and effective sample sizes for analysis of amino acids and hormones following outlier removal are respectively provided in Supplementary Tables S2 and S4. The log2 fold change of each compound was compared between the two conditions within each tissue using Wilcoxon’s rank-sum test as normality could not be assumed for all compounds. Raw p-values were corrected for multiple testing performed on amino acids and phytohormones within each tissue according to Benjamini and Hochberg [89]. The complete output of statistical tests and p-value adjustments pertaining to amino acids and hormones are respectively provided in Supplementary Tables S3 and S5.

## Results

### Irradiation induced altered meristem morphology

**Fig. 1:**
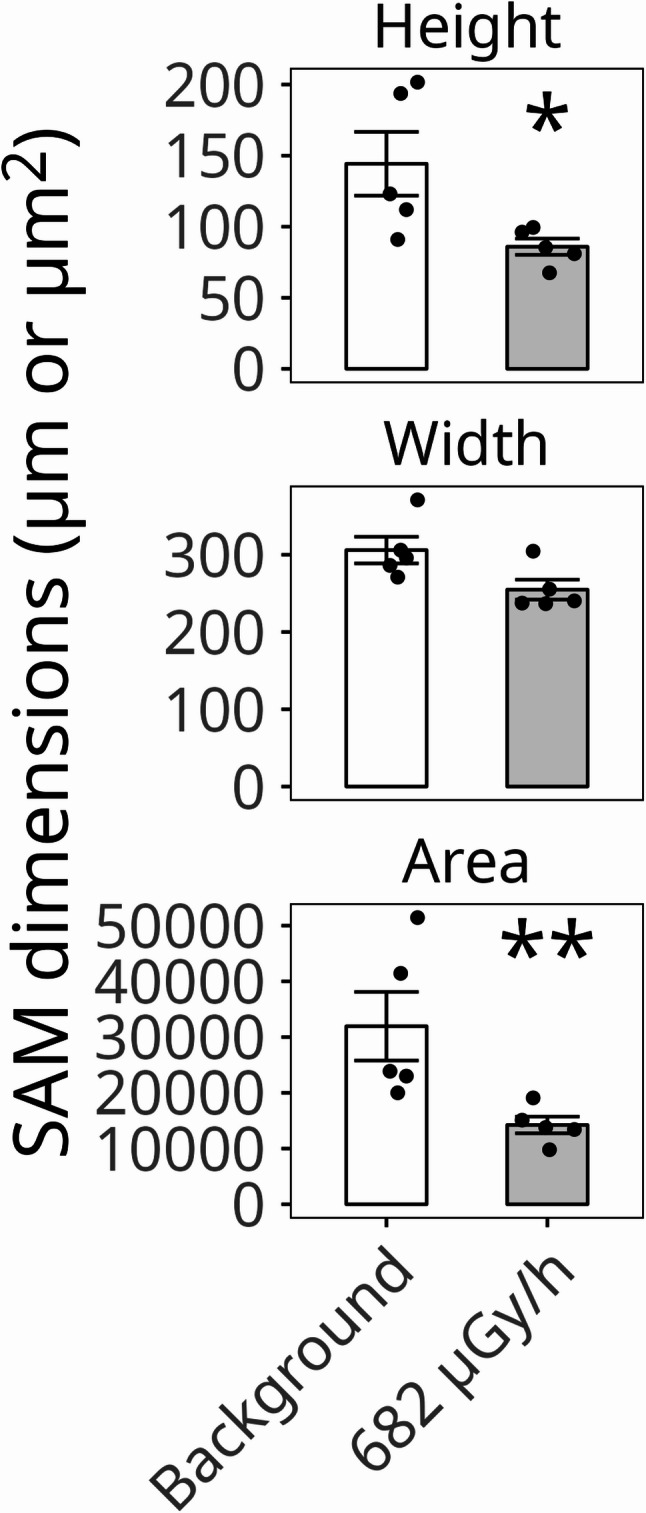
Effect of 10 weeks of chronic gamma irradiation at 682 µGy/h on height (µm), width (µm), and area (µm^2^) of the SAM of young pine seedlings. Data of individual biological replicates (*n* = 5) are represented as solid points, while bars and error bars indicate mean ± standard error. Significant differences between the two conditions were assessed using Wilcoxon’s rank-sum test and are indicated with * (*p* < 0.05) and ** (*p* < 0.01)

We investigated the effect of a 10-week continuous gamma irradiation at a dose rate of 682 µGy.h^− 1^ on the SAM of young pine seedlings, and measurements of the apical dome in longitudinal sections (Supplemental Fig. 1) revealed significant alterations of meristem morphology upon radiation exposure (Fig. 1). Notably, the height of the apical dome was significantly smaller in the treatment group compared to unexposed seedlings (*p* < 0.05). The width of the apex was also slightly decreased by the treatment (Fig. 1). Overall, irradiation treatment resulted in a significant decrease in total meristem area (*p* < 0.01). Interestingly, there were no visually apparent signs of necrosis or damage in the SAM of irradiated seedlings. Despite the changes in shoot meristem morphology, there were no significant effects of the radiation treatment on the shoot phenotype at the macroscopic scale; neither the shoot fresh weight nor the formation and outgrowth of buds and branches were affected by the treatment (Supplemental Fig. 2).

### Targeted metabolomics analysis suggests tissue-specific changes to phytohormone and amino acid abundances

**Fig. 2:**
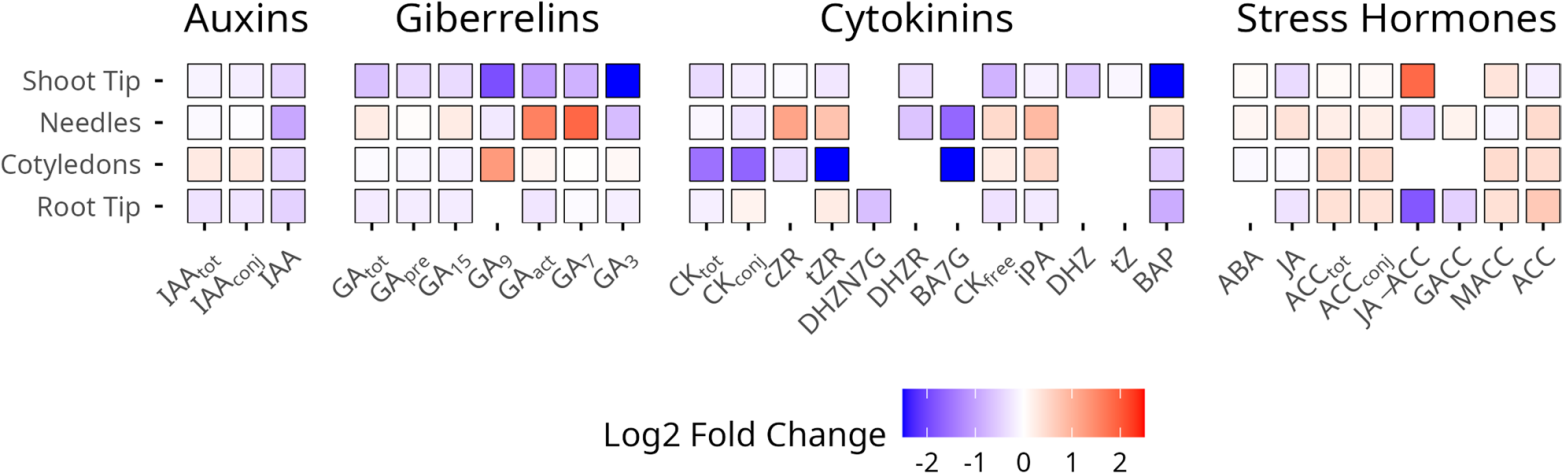
Effect of 10 weeks of chronic gamma irradiation at 682 µGy/h on the abundance of phytohormones in different tissues of young pine seedlings. Data are presented as the mean log_2_ fold change in the treatment condition relative to the mean concentration in the control. Prior to outlier removal, *n* = 6 for shoot tips and *n* = 5 for other tissues. Effective sample sizes following outlier removal are given in Supplementary Table S4. Missing tiles indicate that no fold change was calculated due to concentrations being below the limit of detection. Differences in hormone levels between treated and control samples were assessed using Wilcoxon’s rank-sum test followed by Benjamini-Hochberg correction for the multiple comparisons made within each tissue. No statistically significant differences were retained following p-value adjustment. ACC: 1-aminocyclopropane-1-carboxylic acid. MACC: malonyl-ACC. GACC: glutamyl-ACC. JA-ACC: jasmonyl-ACC. ACCconj: sum of ACC conjugates. ACC_tot_: sum of free and conjugated ACC. JA: jasmonic acid. ABA: abscisic acid. BAP: benzylaminopurine. tZ: *trans*-zeatin. DHZ: dihydrozeatin. iPA: isopentenyladenine. CK_free_: sum of free CKs. BA7G: BAP-7-glucoside. DHZR: DHZ riboside. DHZN7G: DHZ-N7-glucoside. tZR: *trans*-zeatin riboside. cZR: *cis*-zeatin riboside. CK_conj_: sum of conjugated CKs. CK_tot_: sum of free and conjugated CKs. GA: gibberellic acid. GA_act_: sum of biologically active GAs (GA3 and GA7). GA_pre_: sum of active GA precursors (GA9 and GA15). GA_tot_: sum of active and precursor GAs. IAA: indole-acetic acid. IAA_conj_: conjugated IAA. IAA_tot_: sum of free and conjugated IAA

We quantified the abundance of different auxins, gibberellins, CKs and stress-associated phytohormones or related conjugates (Fig. 2). Specifically, we analyzed the primary auxin indole-acetic acid (IAA) in free and conjugated form, two biologically active GAs (GA3 and GA7), two active GA precursors (GA9 and GA15), several CKs (*trans*-zeatin (*t*Z), dihydrozeatin (DHZ), isopentenyladenine (iPA), benzylaminopurine (BAP)) and CK conjugates (benzylaminopurine-*N7*-glucoside (BA*7*G), DHZ riboside (DHZR), DHZ-*7 N*-glucoside (DHZ*N7*G), *t*Z-riboside (*t*ZR) and *cis*-zeatin riboside (*c*ZR)), abscisic acid (ABA), jasmonic acid (JA), the ethylene precursor aminocyclopropane-carboxylic acid (ACC) in free form and conjugated to malonic acid (MACC), glutamate (GACC) and jasmonic acid (JA-ACC).

Overall, phytohormonal changes in response to radiation exposure were limited, and neither individual nor summed hormone concentrations were significantly affected following p-value adjustment for multiple testing (see Limitations). Nonetheless, a number of observable trends in the response are reported below for the sake of future investigation with larger sample sizes, and may still merit discussion. Most detectable CKs and their conjugates showed a slight decrease in irradiated shoot tips, resulting in a considerable decrease in total cytokinin content (p-adj = 0.12). Similarly, the abundance of GAs also tended to decrease overall after irradiation treatment leading to a decrease in total GA (p-adj = 0.12). The same decreasing trend could also be observed for IAA, both free and conjugated, but the statistical significance of this trend was negligible. A decreasing trend for ACC (p-adj = 0.14) could also be observed in the irradiated shoot tip, while its conjugate form jasmonyl-ACC was seemingly increased (p-adj = 0.11). Interestingly, a complete reversal of this trend could be observed in the young needles, with increasing trends for JA (p-adj = 0.19) as well as ACC (p-adj = 0.14) and a decreasing trend for JA-ACC (p-adj = 0.12). In contrast to the shoot tip, CK and GA levels were not uniformly affected in the young needles. The strongest trends for these phytohormones were an increased abundance of the cytokinin *c*ZR (p-adj = 0.19) and the bioactive GAs (p-adj = 0.14), the latter being driven by an increasing of GA_7_ (p-adj = 0.14). Similar to the shoot tip, auxin content in the needles again showed a decreasing tendency but with minimal statistical significance. In the cotyledons, there was an overall tendency of CK levels to decrease after irradiation, with iPA forming a notable exception. The magnitude of changes to phytohormone levels in the root tip were overall even more limited compared to other tissues and had negligible statistical significance even prior to p-value adjustment. With the exception of the above mentioned tendencies of ACC and *c*ZR in the young needles, all observed trends depended on the exclusion of outliers from the statistical analysis.

**Fig. 3:**
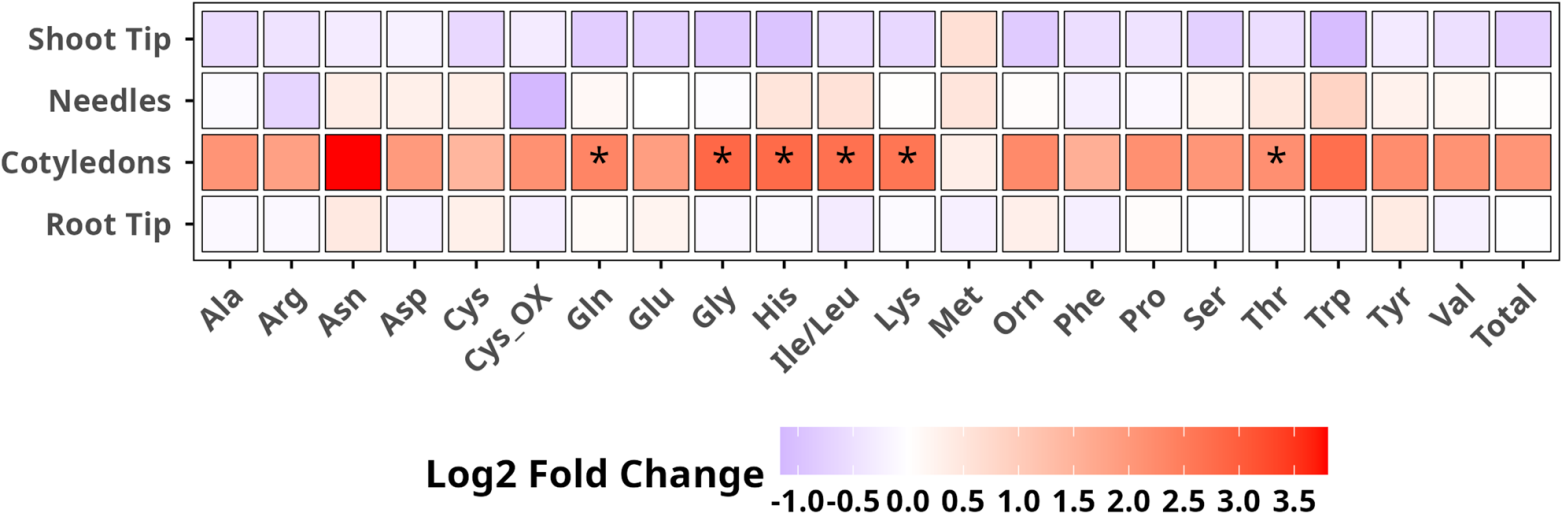
Effect of 10 weeks of chronic gamma irradiation at 682 µGy/h on the abundance of amino acids in different tissues of young pine seedlings. Data are presented as the mean log_2_ fold change in the treatment condition relative to the mean concentration in the control. Prior to outlier removal, *n* = 6 for shoot tips and *n* = 5 for other tissues. Effective sample sizes following outlier removal are given in Supplementary Table S2. Differences in amino acid levels between treated and control samples were assessed using Wilcoxon’s rank-sum test followed by Benjamini-Hochberg correction for the multiple comparisons made within each tissue, and significan differences are indicated as * (p-adj < 0.05). Cys_OX represents oxidized Cys (cystine)

We quantified the abundance of all proteogenic amino acids as well as cystine (oxidized Cys) and Orn (Fig. 3). Irradiation caused an considerable increase in amino acid concentrations in the cotyledons, with significantly affected amino acids being up to 4 times more abundant in the treatment group. Consequently, total amino acid content (calculated as the sum of all amino acid concentrations) was markedly higher in the cotyledons of irradiated seedlings than in controls, although with marginal statistical significance (p-adj = 0.076). An opposite trend could be observed in the shoot tip, as essentially all amino acids were slightly reduced in this tissue after radiation exposure (Fig. 3). Interestingly, Met seemed to resist the otherwise global trends present in the data, being the only amino acid to be upregulated instead of downregulated in the shoot tip, while also being only minimally more abundant among the large, global increase in amino acid concentrations in the cotyledons. Indeed, the mean logFC value of Met was designated as a statistically significant outlier (Grubbs’ outlier test, *p* < 0.05) among the other amino acids in both tissues. Identical results to those described above were obtained when outliers were included in the statistical analysis, although a lower amount of amino acids were significantly affected in the cotyledons.

We similarly observed a distinct shift in Met concentration in a pilot experiment featuring identical culture conditions, irradiation treatment and metabolomics analysis but with the shoot tip included with the young needle sample (Supplementary Fig. 3). After metabolomic analysis of cotyledon and young shoot (shoot tip + young needles) samples and calculation of cotyledon/shoot concentration ratios, we observed a significant increase in this ratio (i.e. increased abundance in the cotyledon relative to the young shoot) for various amino acids, while Met was the only amino acids where this ratio decreased significantly.

### RT-qPCR analysis reveals radiation-induced alteration of hormone-related gene expression

**Fig. 4:**
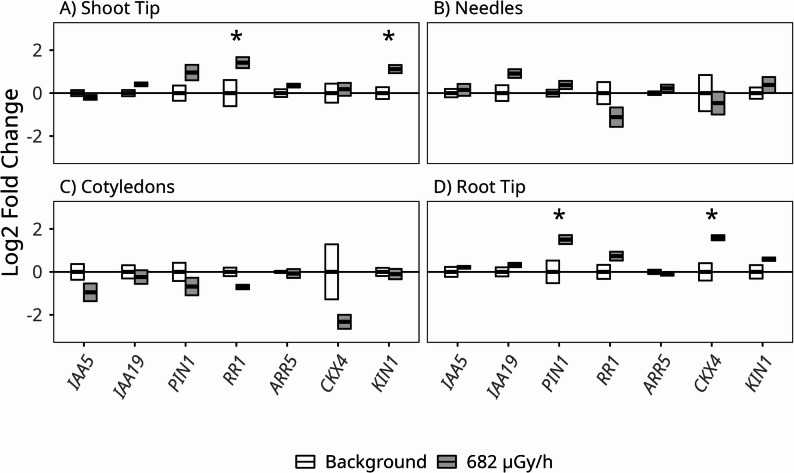
Effect of 10 weeks of chronic gamma irradiation at 682 µGy/h on the expression of selected hormone-related genes in different tissues of young pine seedlings. Data are expressed as a log_2_ fold change relative to the mean of the control. Cross bars indicate the mean ± standard error. Significant differences between the two conditions (*n* = 5 per condition) were assessed using Wilcoxon’s rank-sum test and are indicated with * (*p* < 0.05). *IAA5*, *INDOLE-3-ACETIC ACID INDUCIBLE 5*. *IAA19*, *INDOLE-3-ACETIC ACID INDUCIBLE 19*. *PIN1*, *PIN-FORMED 1*. *ARR5*, *ARABIDOPSIS RESPONSE REGULATOR 5*. *RR1*, *RESPONSE REGULATOR 1*

To further investigate the effects of chronic irradiation on hormone homeostasis in pine seedlings, we performed targeted gene expression analysis on a panel of hormone-related genes using RT-qPCR. Auxin-related genes included *INDOLE-3-ACETIC ACID INDUCIBLE 5* (*IAA5*) and *INDOLE-3-ACETIC ACID INDUCIBLE 19* (*IAA19*), two canonical members of the Aux/IAA protein family which is crucial to the auxin signaling pathway, and *PIN-FORMED 1* (*PIN1*), a canonical auxin transporter [90]. We also analysed three genes related to CK: *RESPONSE REGULATOR 1* (*RR1*, characterized in *P. pinea* [87]) and *ARABIDOPSIS RESPONSE REGULATOR 5 (ARR5*), two type-A response regulators that are involved in CK signaling and shoot development [87, 91], and *CKX4*, a CK oxidase/dehydrogenase responsible for irreversible inactivation of CK and CK conjugates [24]. Lastly, we measured the transcription of *KIN1* [92], a general stress-inducible gene well known to be upregulated by ABA as well as numerous abiotic stressors such as drought, cold and osmotic stress [92–95]. Similarly to the metabolomic data, we observed a highly tissue-specific response to irradiation at the transcriptional level (Fig. 4). In the shoot tip of irradiated seedlings, *RR1* was upregulated by 1.42 log_2_ fold while *KIN1* was upregulated 1.12 log_2_ fold, suggesting a stress-related transcriptional response in this tissue with possible involvement of CK signaling. Meanwhile, no genes were significantly affected in the surrounding young needles nor in the cotyledons. In the root tips, we observed a radiation-induced increase in the expression of *PIN1* (1.5 log_2_ fold change) as well as *CKX4* (1.59 log_2_ fold change), implying that the treatment may have affected auxin transport and CK degradation in the root.

## Discussion

### Altered meristem morphology coincides with a decreasing trend in growth-promoting hormones in the shoot tip

Phytohormonal changes in the shoot tip of pine seedlings chronically exposed to gamma radiation at 682 µGy/h for 10 weeks were characterized by a general trend towards decrease of the growth-promoting hormones CK and GA (Fig. 2). Cytokinin is well-known as a key hormone in the SAM, mediating cell proliferation and stem cell maintenance in the meristem [25]. Conversely, low GA concentrations in the SAM are necessary for preserving an undifferentiated state of the meristem, whereas elevated GA levels stimulate cell division and expansion during SAM development and organ initiation [31, 96, 97]. Our results agree with a general tendency of CK concentrations to decrease in shoot tissue during chronic exposure to abiotic stressors [98, 99], as for example has been observed in pine species during long-term drought treatment [68]. Similarly, various abiotic stresses are known to limit the GA signal [33, 100], which also serves to limit shoot growth during less favorable conditions [101]. Hence, the overall decrease in growth-promoting hormones observed in this study (Fig. 2) is consistent with a stress response in the shoot tip in which SAM proliferation may be limited during radiation stress, potentially redirecting resources towards stress acclimation. Furthermore, the GA signaling pathway connects growth with stress survival via GA-repressed DELLA proteins, which not only inhibit growth but also promote oxidative stress tolerance by upregulation of antioxidative enzymes [102]. The observed tendency towards decreasing GA and CK levels in shoot tips coinciding with the observed decrease in SAM dimensions agrees with the fact that shoot meristem size is known to correlate positively with the abundance of both GAs [32, 96, 103, 104] and CKs [25, 105] in the shoot tip, but whether these hormonal changes are causally related to the decrease in SAM size remains to be investigated.

The significant meristematic changes coincided with an unaffected macroscopic phenotype, two findings whose discrepancy is striking but which are not necessarily incompatible. One possibility is that, in slow-growing organisms such as conifer seedlings, a 10-week period may be sufficient to induce structural and hormonal alterations at the tissue level while being too short for these changes to propagate into detectable differences in shoot biomass, bud number, or branching. Shoot development integrates growth over extended developmental windows and is additionally buffered by compensatory processes involving, for instance, developmental priming and cell wall remodeling [106–108]. The absence of a macroscopic phenotype at this stage therefore does not preclude biologically meaningful meristematic changes, but does underscore the importance of investigating organismal responses at multiple scales of organization and at different time scales.

### Radiation exposure may affect canonical stress hormones and potentially involve tissue-specific modulation of jasmonyl-ACC abundance

Contrary to the CKs and GAs, the abundance of ACC did not reflect a stress response in the shoot tip upon 10 weeks of low-dose radiation exposure, as the abundance of this ethylene precursor showed a decreasing tendency, while in needles it showed an opposite response (Fig. 2). The latter agrees with metabolomic studies in chronically stressed conifers, which indicated a significant increase in ACC levels in needles of drought-stressed Scots pine seedlings [66], while salt stress induced a two-fold increase in the abundance of ACC in the needles of *P. tabuliformis* seedlings [109]. In other contexts, ACC accumulation has been associated with upregulation of ethylene-responsive transcription factors that improve stress tolerance [109]. ACC accumulation during abiotic stress is, at least partially, subject to transcriptional regulation, as such conditions commonly trigger an upregulation of ACC/ethylene biosynthetic enzymes, as well as their upstream transcription factors [42]. To our knowledge, this is the first report of gamma radiation effects on ACC abundance in a conifer species.

Similar to ACC, the abundance of JA seemed to be affected by ionizing radiation in a tissue-specific manner, showing an increasing trend in the young shoot tissue but not in the shoot tip (Fig. 2). Various abiotic stressors lead to the accumulation of JA in plants, which may play a role in the management of the resulting oxidative stress [48, 110]. Furthermore, JA helps shift the growth-defense trade-off towards acclimation during stress conditions, thereby promoting stress tolerance [111], including during drought and heat stress in pine species [66, 68, 69]. Jasmonic acid activation through conjugation to isoleucine has been linked to improved drought tolerance and the alleviation of oxidative stress [112], the latter being particularly relevant for coping with radiogenic ROS, though this conjugation was not measured in the present study.

An interesting pattern emerges when considering the abundance of both JA and ACC as well as their conjugation form, JA-ACC. The tendency for decreased abundance of the stress-associated ethylene precursor ACC in the shoot tip coincided with a trend of increased abundance of its jasmonyl form (Fig. 2). Hence, irradiation treatment was seemingly associated with an increase in JA-ACC coupling in this tissue. In contrast, a completely opposite effect could be observed in the needles: ACC and JA became slightly more abundant after radiation exposure, while JA-ACC abundance was diminished. Hence, irradiation treatments seemed to have tissue-specific effects on JA-ACC formation.

In *Arabidopsis*, JA-ACC formation has been attributed to jasmonate-conjugating enzymes such as GH3.10 and JASMONATE RESISTANT 1 (JAR1), which are also required for the conversion of inactive JA to its bioactive JA-Ile form [113–115]. However, JA-ACC metabolism has not yet been further elucidated, and the physiological roles of JA-ACC are still unclear. In *A*. *thaliana* roots, JA-ACC treatment mimicked ACC’s growth inhibitory effect independently from the JA signaling pathway while requiring ethylene signaling [113], which may have been due to the release of ACC and subsequent conversion to ethylene in the root. More notably, JA-ACC has been hypothesized to integrate the JA and ACC pathways by sequestering the primary inactive precursors of both the ethylene and jasmonate signaling pathways in a single reaction [113]. In this context, JA-ACC formation could hypothetically buffer growth-inhibiting effects associated with low CK and GA levels in the irradiated SAM, while ACC and JA accumulation in the growing needle tissue could possibly involve recruitment from the JA-ACC pool. Alternatively, the observed changes in JA-ACC and corresponding ACC content may potentially result from altered conjugation-mediated JA activation.

Chronic gamma-irradiation of *A*. *thaliana* led to increased abundance of conjugated ACC but not free ACC [73], further highlighting the potential role of ACC conjugation in the plant radiation response, although the functional significance of this remains to be established. Due to the limited understanding of JA-ACC’s role and significance in plant physiology and a complete lack of studies involving JA-ACC in conifers, the potential importance and physiological function of this compound in the radiation response of pine seedlings remains uncertain. Deep metabolomic profiling of the JA pool, coupled with a transcriptional analysis of ethylene and jasmonate-related genes (e.g. *JAR1* and *GH3.10*), could help clarify JA-ACC conjugation patterns during chronic radiation exposure and shed light on its potential involvement in the response.

Finally, in literature some indirect evidence of radiation-induced changes to ethylene homeostasis has been reported coming from the acute irradiation exposure of pine trees immediately following the Chornobyl accident. This exposure caused widespread senescence of pine needles [116], a phenomenon mediated by ethylene [42]. However, it is clear that the chronic irradiation utilized in the present study shows a more nuanced response in ethylene metabolism. Interestingly, although we did not detect variation in auxin levels after 10 weeks of exposure in pine seedlings, accumulation of this hormone has been reported in adult Scots pine growing under chronic radiation exposure in the Chornobyl Exclusion Zone [71], suggesting that radiation-induced hormone level modulations may also depend on the plant’s developmental stage.

### Potential modulation of specific cytokinin and gibberellin levels in irradiated needles

One of the notable phytohormonal trends in the young needles of irradiated seedlings was a considerable increase in the cytokinin conjugate *c*ZR. The presence of a measurable increase in shoot *c*ZR content in absence of detectable *c*Z in the root tip indicates this response is likely due to local *c*Z metabolism in the shoot (Fig. 2). The physiological roles and regulatory mechanisms pertaining to *c*Z are not fully understood, but its increase after radiation exposure is in agreement with current knowledge as this CK and its derivatives are known to increase in shoot tissues under various stress conditions and during periods of growth limitation [117]. Despite its weak activity, the high stability of cZ allows it to modulate CK signaling under stress by competing for metabolic enzymes and receptors, a process likely supported by stress-triggered tRNA prenylation and degradation which serves as the primary biosynthetic pathway [117, 118]. Both free and conjugated *c*Z accumulates in drought-stressed Scots pine seedlings and saplings [66, 67], and after heat stress in somatic embryos of Aleppo pine (*P. halepensis*) after heat stress [119], possibly indicating a conserved response across different stressors and tissues. This may be related to the relative importance of *c*Z in pine growth, as over 80% of zeatin-type CKs measured in *P. sylvestris* shoot tissue were previously found to be of the *cis* confirmation [120], although in our study the *trans* zeatin forms were more abundant in the shoot tissue than the *cis* forms (Supplemental Table 1). Interestingly, while the different zeatin isomers were not distinguished, an increase in zeatin content has been observed in chronically irradiated adult pines needles in Fukushima [70], further highlighting the importance of zeatin-type CKs in the pine radiation response. It must be noted that CK ribosides such as *c*ZR are considered inactive transport forms that must be converted to free bases for CK signaling to occur [121], and because the free *c*Z was below the LOD in our analysis, we cannot conclude that irradiation modulated the concentration of bioactive *c*Z. Nonetheless, some CK ribosides bind CK receptors without activating them and can compete with active ligands [121], hence the radiation-induced increase in conjugated cZ may still modulate signaling, although this remains to be investigated.

Another trend observable in the irradiated young needles was an increased abundance of bio-active GA (Fig. 2). This is a surprising finding, given that GA concentrations tend to decrease during stress conditions, resulting in the accumulation of the growth-inhibitory DELLA proteins that act downstream of GA [33, 100, 101]. However, the same trend has been reported for needles of Japanese red pine trees growing in radioactively contaminated areas of Fukushima [70]. Such a GA increase may hypothetically temper the effect of the ACC accumulation and potential ethylene production upon radiation exposure, as ethylene generally antagonises GA-mediated growth [122]. Alternatively, an as-yet unknown mechanism could integrate ACC and JA with GA via positive feedback. Ethylene can act synergistically with GA to induce elongation under specific conditions, such as during hypocotyl elongation of *A. thaliana* [123] and submergence-induced anoxia stress in *Oryza sativa* [100]. The basis of the radiation-induced GA increase remains unclear and warrants further investigation. Given the role of GA in cell elongation, it will be important to test whether higher active GA after radiation exposure is reflected phenotypically through an increase in needle length.

### Global amino acid changes implies altered protein metabolism

Despite no significant change in the total amino acid content, all individual amino acid concentrations increased in the cotyledons (Fig. 3). This global radiation-induced change implies an effect on protein metabolism rather than effects at the amino acid level, which is in agreement with multiple transcriptome analyses indicating differential expression of genes involved in protein degradation and protein synthesis upon irradiation treatment in plants [124–127]. Cotyledons are inherently temporary organs, providing nutrients and photosynthesis to germinating seedlings, followed by senescence with decreased protein synthesis, increased protease-mediated protein degradation and nutrient reallocation to growing tissues [60, 128–130]. Numerous abiotic stressors can induce senescence and the associated proteolysis [131]. Hence, the observed increase in cotyledon amino acid content (Fig. 3) could potentially be related to accelerated cotyledon senescence triggered by radiation exposure, a hypothesis which is supported by the tendency of most CKs to decrease in the cotyledons upon irradiation as CK acts as a senescence repressor [26]. The global amino acid increase in the cotyledons is in stark contrast to the global pattern observable in the shoot tip (Fig. 3). These findings highlight a need for further research into cotyledon senescence, protein turnover and resource allocation during chronic radiation exposure of pines.

### An integral role for methionine in the pine radiation response

While irradiation treatment had an overall effect on amino acid content in both the cotyledons and the shoot tip, Met clearly behaved differently (Fig. 3; Supplemental Fig. 3). In contrast to the other amino acids, Met only minimally increased in the cotyledons. Furthermore, Met was the only amino acid to increase in the shoot tissue, where all others showed a decreasing trend. In both cases, Met’s mean logFC value was identified as a statistically significant outlier within the tissue. These data illustrates a tight regulation of the Met pool and an integral role of this amino acid during radiation exposure. Methionine is the direct precursor to S-adenosyl methionine, which is a crucial precursor to ACC and therefore ethylene [132]. Hence, the accumulation of Met in the shoot tips in the presence of amino acid depletion may be connected to the decreased abundance of ACC in this tissue. The aberrant behavior of Met may also relate to its importance for oxidative stress response, as we have previously shown that a 10-week low-dose gamma irradiation induces oxidative stress and affects the metabolism of the important antioxidant glutathione in pine seedlings [77]. Several studies indicate that Met and glutathione compete directly for their shared precursor Cys [56], and glutathione oxidation can shift the Cys flux from Met biosynthesis towards glutathione [133]. Furthermore, both free and protein-bound Met can act as a ROS scavenger through reversible oxidation of the sulfur residue [134], while Met can alleviate oxidative stress and improve abiotic stress tolerance in various plant species [135, 136], Met can also act as a ROS-mediated stress signal independently from ethylene under stress conditions [136]. Further study is required to uncover the exact roles of Met in the pine radiation response.

### Radiation induces a distinct and tissue-specific transcriptional response

We observed a significant upregulation of the stress-associated transcript *KIN1* in the shoot tip (Fig. 4), implying that the observed trends towards growth-promoting hormones (CK, GA) in that tissue may be the result of a stress response due to irradiation, despite the absence of accumulating stress hormones (e.g. ACC, JA, ABA). Interestingly, the increased expression of this canonically ABA-inducible protein did not coincide with an accumulation of ABA in this tissue (Fig. 2), which could potentially be explained by a transient modulation of ABA levels prior to our analysis or by *KIN1* upregulation occurring via an alternative pathway. KIN1 is a member of the COLD REGULATED protein family, under control of the C-repeat/dehydration-responsive element (CRT/DRE) promoter motif during abiotic stresses [93]. Ethylene is generally acknowledged as a negative regulator of the CRT/DRE pathway, as evidenced in different plant species [137–139]. Hence, the tendency towards decreased abundance of the ethylene precursor ACC observed in our metabolomic analysis after irradiation treatment (Fig. 2) could reflect a transient decrease of ethylene levels, consistent with upregulation of *KIN1*. Furthermore, the *KIN1* expression pattern also correlates with the decrease in GA content in the shoot tip after irradiation treatment, as CBF/DREB transcription factors have been shown to decrease GA abundance [140, 141] and increase GA degradation [140].

We also observed a significant upregulation of the CK-related *RR1* in the shoot tip after irradiation treatment (Fig. 4). This is a remarkable result in view of the trending decrease in CKs in this tissue due to irradiation (Fig. 2), as type-A response regulators such as *RR1* are well-known to be regulated positively by CKs [87, 142, 143]. Interestingly, the expression of several type-A response regulators is negatively regulated by ethylene signaling in *A*. *thaliana* [139], meaning the upregulation of *RR1* correlates with the observed decrease in ACC in the shoot tip, similar to *KIN1*. The radiation-induced upregulation of the CK-responsive *RR1* in presence of a decreased CK pool may indicate a compensatory mechanism, but the exact pathways leading to this phenomenon require further study.

Although no significant changes were observed in root phytohormone levels (Fig. 2), transcriptional changes in this tissue suggest that chronic irradiation may affect auxin transport and cytokinin metabolism in the root, which we hypothesize may indicate reprogramming of lateral root formation. We observed a significant upregulation of *PIN1* (Fig. 4). This gene encodes a canonical polar auxin transporter involved in lateral root formation by establishing a local auxin maximum in lateral root founder cells [144–146], which occurs within 1 cm of distance from the root apex, the region we sampled [147]. This finding contrasts previous studies indicating that *PIN1* expression in roots is decreased by various types of abiotic stress [148–151] but is in agreement with *PIN1* upregulation observed in *A. thaliana* root samples during induction of lateral root initiation [144]. The upregulation of the CK-degrading enzyme *CKX4* in root tips is also consistent with conditions that could promote lateral root formation. Cytokinins inhibit root growth and lateral root formation by suppressing localized *PIN1* upregulation and limiting cell cycle progression [144, 152]. Therefore, a CKX4-mediated increase in CK degradation could also promote lateral root development. Enhanced *CKX* expression has been linked to increased lateral root growth development in multiple plant species [105, 153, 154]. We did not detect a significant decrease in CK abundance despite significant *CKX4* upregulation, but it may be noted that CKX do not affect all CKs equally, and different CKX preferentially degrade different CKs in different plant species [155–159]. Interestingly, recovery after chronic irradiation at a dose rate of 1 mGy/h has previously been shown to result in increased lateral root formation in *Picea abies* [160], supporting the idea that chronic exposure to low-dose ionizing radiation can promote lateral root formation in conifer species. Naturally, phenotypical analysis of pine seedling roots after chronic radiation exposure is required to explore this hypothesis. 

### Limitations of the study

The present study employs a single dose rate (682 µGy/h) and a single terminal sampling time point (10 weeks). While this design is appropriate for characterizing a limited snapshot of the metabolic and transcriptional state of seedlings under this particular exposure regime, it does not permit inference of dose-response relationships, nor does it allow the temporal order of the observed responses to be determined. Accordingly, the sequence in which the various organ-specific changes arise, such as whether the reduction in SAM growth preceded or followed the supposed metabolomic changes, remains unknown. Additionally, the stated dose rate was measured as air kerma at shoot level, and detailed organ-level dosimetry was not performed. Depending on individual differences in growth and morphology and radiation shielding associated with the liquid medium of our hydroponic culture setup, the actual dose rate experienced by the root tip may have been considerably lower in the root tips compared to the shoot tissues, which may explain the limited metabolomic response in this tissue. It should also be noted that, with the exception of *RR1*, the genes analysed were identified in pine by sequence homology to *Arabidopsis*, and their functional roles in pine tissues have not been directly validated. Interpretations of their expression changes therefore assume conservation of gene function, which remains to be confirmed.

Another considerable limitation concerns the statistical resolution of the phytohormonal data. No individual hormone or summed hormone class reached statistical significance following Benjamini-Hochberg correction for multiple testing. This outcome is at least partly attributable to the inherent constraints of the study design: with *n* = 5–6 biological replicates per condition, the Wilcoxon rank-sum test yields a minimum achievable two-tailed p-value of approximately 0.008–0.002. Following correction across the 20–25 detectable hormone comparisons performed within each tissue, even the most extreme possible rank-sum outcome produces an adjusted p-value near or above the 0.05 threshold. Consequently, the design is inherently limited in its capacity to yield FDR-corrected significance for hormonal endpoints, and the absence of significant results should not be interpreted as evidence of no effect. The directional consistency observed across biologically related metabolites, such as the majority of detectable CK species trending in the same direction within a given tissue, lends some biological plausibility to the observed patterns beyond what individual adjusted p-values convey. The phytohormonal findings of this study are therefore best regarded as hypothesis-generating observations, motivating future work with larger sample sizes or complementary analytical approaches better suited to resolving small-magnitude hormonal changes under these conditions.

## Conclusion

**Fig. 5:**
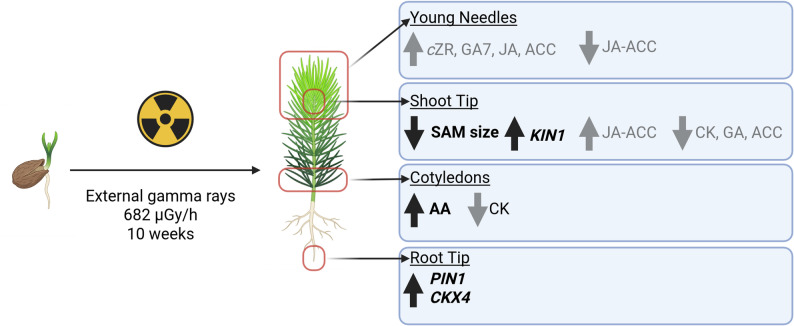
Summary of organ-specific metabolomic and transcriptional responses of *Pinus sylvestris* seedlings to chronic gamma irradiation. *Pinus sylvestris *seedlings were exposed to continuous external gamma irradiation from ¹³⁷Cs sources at a dose rate of 682 µGy/h for 10 weeks under controlled laboratory conditions. The figure summarises key findings from targeted metabolomic analysis of phytohormones and amino acids, microscopic analysis of the shoot apical meristem, and RT-qPCR-based gene expression analysis across four tissues. Up arrows (↑) and down arrows (↓) indicate an increase or decrease, respectively, in the irradiated condition relative to the control. Black arrows and bold text indicate statistically significant changes. Grey arrows and text indicate directional trends that did not reach statistical significance after correction for multiple testing; these are reported as hypothesis-generating observations. Gene names are shown in italics. AA, free amino acids; ACC, 1-aminocyclopropane-1-carboxylic acid (ethylene precursor); CK, total cytokinins; CKX4, CYTOKININ OXIDASE/DEHYDROGENASE 4; *c*ZR, *cis*-zeatin riboside; GA, total gibberellins; GA7, gibberellin A7; JA, jasmonic acid; JA-ACC, jasmonyl-1-aminocyclopropane-1-carboxylic acid; *PIN1*, PIN-FORMED 1; *RR1*, RESPONSE REGULATOR 1; SAM, shoot apical meristem

Our study revealed an organ-dependent pattern of morphological, metabolomic and transcriptional changes in the radiosensitive plant *P. sylvestris* in response to chronic gamma irradiation during early growth stages (Fig. 5). A reduction in SAM size coincided with a localized tendency toward decrease of growth-promoting CKs and GAs, potentially indicating a stress-associated suppression of meristematic development. These findings are underscored by a local upregulation of the stress-inducible gene *KIN1*. A trend toward accumulation of stress-associated hormones in young needle tissue were also suggestive of a localized stress response. Concurrently, the global increase of amino acids and decreased CK abundance in cotyledons are consistent with accelerated senescence, potentially allowing resource reallocation towards stress acclimation. Our targeted gene expression analysis furthermore reveals a new dimension to the pine radiation response, as the significant upregulation of *PIN1* and *CKX4* (consistent with altered auxin transport and cytokinin catabolism) could potentially indicate root architectural remodeling, though this is yet to be quantified. Overall, these organ-dependent changes are consistent with a growth-defense trade-off and emphasize the importance of a multi-tissue perspective when assessing the metabolic impacts of chronic radiation, but also highlight the need for more in-depth investigation into these phenomena.

## Supplementary Information

Supplementary Material 1.

Supplementary Material 2.

Supplementary Material 3.

## Data Availability

The datasets used and/or analysed during the current study are available from the corresponding author on reasonable request.
