# Supplementary material for: Targeted metabolomic and gene expression analysis reveals an organ-specific response to chronic ionizing radiation exposure during Pinus sylvestris seedling development
Source: BMC Plant Biol. 2026 Jun 4;26:1337. doi: 10.1186/s12870-026-09149-7 (PMC13455340; doi:10.1186/s12870-026-09149-7)
Supplement: Supplementary file 1 [file 12870_2026_9149_MOESM3_ESM.pdf]

A. Appendix

A.1 Heavy labelled tracers

- 50 pmol d-GA_1, d-GA_8, d-GA_9, d-GA_{15}, d-GA_{19}, d-GA_{20} (OlchemIm, Olomouc, Czech Republic)

- 300 pmol 3-[13C6]indolylacetic acid ([13C6]-IAA) (OlchemIm, Olomouc, Czech Republic)

- 400 pmol (S)-5-[2H6](1-hydroxy-2,6,6-trimethyl-4-oxocyclohex-2-en-1-yl)-3-methyl-(2Z,4E)-pentadienoic acid ([2H6]-ABA) (OlchemIm, Olomouc, Czech Republic)

- 300 pmol (±)-3-oxo-2-pentyl-cyclopentane-1-acetic acid (DHJA) (OlchemIm, Olomouc, Czech Republic). Add Oasis® HLB Sorbent (Waters, Deerfield, IL, USA)

- 1200 pmol [2H4]1- aminocyclopropaneecarboxylic acid ([2H4]-ACC) (OlchemIm, Olomouc, Czech Republic)

- 1200 pmol 2- amino-[2H3]4-methylsulfanylbutanoic acid ([2H3]-Met) (Sigma-Aldrich, Saint-Louis, MO, USA)

- 50 pmol 10-6 M d-ICK (d-DHZR, d-DHZ, d-DHZ-N9-G, d-Z-N7-G, d-iPG, d-iPA, d-iP)

- 50 pmol 10-6M d-ACK (d-BA, d-BAR, d-BA(9)G, ^{13}C-o-T, ^{15}N-mT)

A.2 Appendix Tables

Table A.1: ABA, JA and IAA metabolites with corresponding transitions and parameters for UPLC-MS/MS measurements. ABA = abscisic acid; JA = jasmonic acid, DHJA = 9,10-dihydrojasmonic acid; IAA = indole-3-acetic acid; N/A = not available.

Metabolite	Protonated molecular ion (Da)	Product ion (Da)	Retention time (min)	Cone voltage (V)	Collision energy (eV)	Internal standard	Correction
**ABA**	420	349	3.50	28	18	[2H6]-ABA	N/A
**ABA**	420	304	3.50	28	24	[2H6]-ABA	N/A
**[2H6]-ABA**	426	355	3.50	28	18	N/A	N/A
**[2H6]-ABA**	426	310	3.50	28	24	N/A	N/A
**JA**	366	295	3.75	26	18	DHJA	N/A
**DHJA**	368	297	3.90	25	18	N/A	N/A
**IAA**	331	260	3.45	27	14	[13C6]-IAA	N/A
**IAA**	331	215	3.37	28	18	[13C6]-IAA	N/A
**[13C6]-IAA**	337	221	3.47	28	18	N/A	N/A

Table A.2: Gibberellin-linked metabolites with corresponding transitions and parameters for UPLC-MS/MS measurements. GA = gibberellic acid/gibberellin; N/A = not available.

Metabolite	Protonated molecular ion (Da)	Product ion (Da)	Retention time (min)	Cone voltage (V)	Collision energy (eV)	Internal standard	Correction
**GA1**	504	433	2.72	42	22	[2H2]-GA1	N/A
**[2H2]-GA1**	506	435	2.72	42	22	N/A	N/A
**GA3**	502	431	2.68	42	22	[2H2]-GA1	0.8637
**GA4**	488	417	4.53	42	22	[2H2]-GA9	0.4809
**GA5**	486	415	3.56	42	22	[2H2]-GA20	1.5472
**GA7**	486	415	4.48	42	22	[2H2]-GA9	0.3866
**GA8**	520	449	1.74	42	22	[2H2]-GA8	N/A
**[2H2]-GA8**	522	451	1.74	42	22	N/A	N/A
**GA9**	472	401	5.17	42	22	[2H2]-GA9	N/A
**[2H2]-GA9**	474	403	5.17	42	22	N/A	N/A
**GA12**	488	417	5.80	42	22	[2H2]-GA15	0.1497
**GA15**	486	415	5.52	42	22	[2H2]-GA15	N/A
**[2H2]-GA15**	488	417	5.52	42	22	N/A	N/A
**GA19**	532	299	4.47	30	24	[2H2]-GA19	N/A
**[2H2]-GA19**	534	301	4.47	30	24	N/A	N/A
**GA20**	488	417	3.64	42	22	[2H2]-GA20	N/A
**[2H2]-GA20**	490	419	3.64	42	22	N/A	N/A

Table A.3: Gradient table for identifying and quantification of aromatic cytokinin-linked metabolites for UPLC-MS/MS. Run time: 8.20 minutes; Solvent A: 1mM ammonium acetate; solvent B: Methanol

Time(min)	Flow Rate	%A	%B	Curve
Initial	0.300	99.9	0.1	Initial
5.00	0.300	72.0	28.0	6
5.50	0.300	0.1	99.9	6
6.00	0.300	1.0	99.9	1
6.10	0.300	99.9	0.1	6
8.00	0.300	99.9	0.1	6

Table A.4: Gradient table for identifying and quantification of isoprenoid cytokinin-linked metabolites for UPLC-MS/MS. Run time: 7.60 minutes; Solvent A: 1mM ammonium acetate; solvent B: Methanol

Time(min)	Flow Rate	%A	%B	Curve
Initial	0.300	95.0	5.0	Initial
0.40	0.300	95.0	5.0	6
3.00	0.300	76.0	24.0	6
5.00	0.300	75.0	25.0	6
6.00	0.300	5.0	95.0	6
6.50	0.300	5.0	95.0	1
6.60	0.300	95.0	5.0	6
7.60	0.300	95.0	5.0	11

Table A.5: Gradient table for identifying and quantification of ACC-linked metabolites for UPLC-MS/MS. Run time: 7.31 minutes; Solvent A: 0.1% FA in water; solvent B: 0.1% FA in ACN

Time(min)	Flow Rate	%A	%B	Curve
Initial	0.400	9.0	91.0	Initial
0.50	0.400	9.0	91.0	6
6.00	0.400	49.0	51.0	6
6.35	0.400	80.0	20.0	1
6.40	0.400	9.0	91.0	6
7.29	0.400	9.0	91.0	11

Table A.6: Gradient table for identifying and quantification of amino acids for UPLC-MS/MS. Run time: 7.31 minutes; Solvent A: 0.1% FA in water; solvent B: 0.1% FA in ACN

Time(min)	Flow Rate	%A	%B	Curve
Initial	0.400	9.0	91.0	Initial
0.50	0.400	9.0	91.0	6
6.00	0.400	49.0	51.0	6
6.35	0.400	80.0	20.0	1
6.40	0.400	9.0	91.0	6
7.29	0.400	9.0	91.0	11

Table A.7: ACC-linked metabolites with corresponding transitions and parameters for UPLC-MS/MS measurements. Dwell: 0.026 sec; delay: auto. ACC = 1-aminocyclopropane-1-carboxylate; N/A = not available.

Compound	Protonated molecular ion (Da)	Product ion (Da)	Cone voltage (V)	Collision energy (eV)	Retention time (min)
**ACC**	102	56	22.0	17.0	
**d4-ACC**	106	60	22.0	17.0	
**Malonyl ACC rt 1.4**	188	142	20.0	14.0	1.4
**Malonyl ACC rt 1.4**	188	170	20.0	9.0	1.4
**Glutamyl ACC rt 0.8**	231	84	18.0	24.0	0.8
**Glutamyl ACC rt 0.8**	231	168	18.0	22.0	0.8
**Jasmonyl ACC rt 3.7**	294	151	25.0	16.0	3.7
**Jasmonyl ACC rt 3.7**	294	276	25.0	15.0	3.7

Table A.8: Amino acids with corresponding transitions and parameters for UPLC-MS/MS measurements. Dwell: 0.022 sec; delay: auto.

Compound	Protonated molecular ion (Da)	Product ion (Da)	Cone voltage (V)	Collision energy (eV)
**Glycine**	76	30	13.0	6.0
**Glycine**	76	48	13.0	6.0
**Alanine**	90	44	10.0	8.0
**GABA**	104	87	17.0	10.0
**Serine**	106	70	23.0	12.0
**Serine**	106	88	23.0	12.0
**Proline**	116	70	20.0	12.0
**Ornithine**	133	70	17.0	11.0
**Asparagine**	133	74	12.0	9.0
**Asparagine**	133	88	12.0	9.0
**Glutamine**	147	84	14.0	10.0
**Glutamine**	147	130	14.0	10.0
**Glutamic Acid**	148	84	14.0	9.0
**Glutamic Acid**	148	130	14.0	9.0
**d3-methionine**	153	107	15.0	14.0
**Histidine**	156	110	23.0	15.0
**Arginine**	175	70	12.0	14.0
**Arginine**	175	130	12.0	14.0
**Norvaline**	118	72	13.0	8.0
**Threonine**	120	74	15.0	15.0
**Cysteine**	122	61	15.0	15.0
**(iso)leucine**	132	86	15.0	15.0
**Aspartic acid**	134	74	15.0	15.0
**Lysine**	147	84	15.0	15.0
**Methionine**	150	104	15.0	15.0
**Valine**	151	72	15.0	15.0
**d3-methionine**	153	107	15.0	14.0
**Phenylalanine**	166	120	15.0	15.0
**Tyrosine**	182	136	15.0	15.0
**Tryptophan**	205	146	15.0	15.0
**Tryptophan**	205	188	15.0	15.0
**Cystine**	241	151	15.0	14.0

Table A.10: Primer sequence and primer efficiency of the primer pairs used for gene expression analysis. Reference genes ACT = Actin; YLS8 = YELLOW-LEAF-SPECIFIC8; PEX4 =Peroxin4. Auxin related genes IAA5 = Auxin/Indole-3-Acetic Acid5, IAA19 = Auxin/Indole-3-Acetic Acid19, PIN1= PIN-FORMED1; Abscisic acid related gene KIN1= serine/threonine protein kinase1; and Cytokinin related genes ARR5 = *Arabidopsis* response regulator5, RR1 = response regulator gene1, CKX4 = cytokinin oxidase4. Efficiency is calculated using the equation: $E = -1+10^{(-1/slope)}$.

Hormone	Gene		Sequence	Efficiency (%)
Reference	ACT	Forward	TGACATGGAGAAGATTTGGC	103.55
		Reverse	CATACATAGCAGGCACATTG	
	YLS8	Forward	AGAATGGCCTGATCCACTGC	102.85
		Reverse	AGGATCGAGCGAGGCAAAAA	
	PEX4	Forward	AGCTTTCCTGCGATGACACA	92.31
		Reverse	GTCACAAAGCGCACTTGAGG	
Auxin	IAA5	Forward	GCCTGGGACAGATCAATAAACTC	103.62
		Reverse	TGAAGTATGGTACACAGAATGCC	
	IAA19	Forward	TCCCTTCAGTCCACCAAAGAC	90.90
		Reverse	CCAGCCAGTCAGCAACAATG	
	PIN1	Forward	TCTCTTCCATCCAGGGACATAAC	94.70
		Reverse	GTGCCAAGATACTCATAGCTGAAC	
Abscisic Acid	KIN1	Forward	TCAACCCGGAAGGTTATCAAGG	94.01
		Reverse	AAGATTGGGATGGTGGAGTTGG	
Cytokinins	ARR5	Forward	AGGCTGCTGTTGCTCCTTTG	92.50
		Reverse	ACCTGTGAAGTTAGCGGATGTG	
	RR1	Forward	TGTCAGAAGGCGCTGAAG	90.53
		Reverse	GTTGGTCCCTGGATCTTCC	
	CKX4	Forward	ATTGAACTGCTCCAGACACC	97.90
		Reverse	CGATCCTGGCCTTCGTTATG	
