# Supplementary material for: Targeted metabolomic and gene expression analysis reveals an organ-specific response to chronic ionizing radiation exposure during Pinus sylvestris seedling development
Source: BMC Plant Biol. 2026 Jun 4;26:1337. doi: 10.1186/s12870-026-09149-7 (PMC13455340; doi:10.1186/s12870-026-09149-7)
Supplement: Supplementary file 2 [file 12870_2026_9149_MOESM1_ESM.docx]

Supplementary Figure 1

Supplementary Figure 2

Supplementary Figure 3
